# Cardiac function and incidence of unexplained myocardial scarring in patients with primary carnitine deficiency - a cardiac magnetic resonance study

**DOI:** 10.1038/s41598-019-50458-9

**Published:** 2019-09-26

**Authors:** Kasper Kyhl, Tóra Róin, Allan Lund, Niels Vejlstrup, Per Lav Madsen, Thomas Engstrøm, Jan Rasmussen

**Affiliations:** 1 Department of Medicine, The National Hospital, Tórshavn, Faroe Islands; 2 The Cardiac MRI Groups, Departments of Cardiology, Rigshospitalet, Copenhagen, Denmark; 3 Centre for Inherited Metabolic Diseases, Departments of Paediatrics and Clinical Genetics, Rigshospitalet, Copenhagen, Denmark; 4 Department of Cardiology, Rigshospitalet, Copenhagen, Denmark; 5 Department of Cardiology, Copenhagen University Hospital, Herlev-Gentofte, Copenhagen, Denmark; 6 Department of Clinical Medicine, Copenhagen University, Copenhagen, Denmark

**Keywords:** Medical research, Cardiovascular biology

## Abstract

Primary carnitine deficiency (PCD) not treated with L-Carnitine can lead to sudden cardiac death. To our knowledge, it is unknown if asymptomatic patients treated with L-Carnitine suffer from myocardial scarring and thus be at greater risk of potentially serious arrhythmia. Cardiac evaluation of function and myocardial scarring is non-invasively best supported by cardiac magnetic resonance imaging (CMR) with late gadolinium enhancement (LGE). The study included 36 PCD patients, 17 carriers and 17 healthy subjects. A CMR cine stack in the short-axis plane were acquired to evaluate left ventricle (LV) systolic and diastolic function and a similar LGE stack to evaluate myocardial scarring and replacement fibrosis. LV volumes and ejection fraction were not different between PCD patients, carriers and healthy subjects. However, LV mass was higher in PCD patients with the severe homozygous mutation, c.95 A > G (p = 0.037; n = 17). Among homozygous PCD patients there were two cases of unexplained myocardial scarring and this is in contrast to no myocardial scarring in any of the other study participants (p = 0.10). LV mass was increased in PCD patients. L-carnitine supplementation is essential in order to prevent potentially lethal cardiac arrhythmia and serious adverse cardiac remodeling.

## Introduction

Primary carnitine deficiency (PCD) not treated with L-Carnitine can lead to sudden cardiac death^1,2^. Patients homozygous for the PCD related c.95 A > G mutation in *SLC22A5* have been shown to be especially vulnerable with regards to potentially lethal cardiac arrhythmia. This genotype is especially prevalent in the small island community of the Faroe Islands^3,4^. Cardiac fibrosis assessed by late gadolinium enhancement (LGE) with cardiac magnetic resonance (CMR) is positively correlated to ventricular tachyarrythmias and SCD in various cardiomyopathies^5–7^. Late gadolinium enhancement (LGE) with cardiac magnetic resonance (CMR) is a marker of myocardial fibrosis of any etiology^8^.

PCD is an autosomal recessive disorder of fatty acid oxidation caused by a dysfunctional organic carnitine (OCTN2) transporter. The transporter is highly expressed in myocardial cells and consequently attains a high plasma-myocardial gradient of carnitine. A dysfunctional OCTN2 transporter reduces this gradient because of a compromised myocardial carnitine transport as well as a result of low plasma carnitine concentration subsequent to excessive renal loss of carnitine. However, carnitine is a vital metabolite for the heart as it is necessary for the transfer of long-chain fatty acids across the inner mitochondrial membrane for beta-oxidation. Apart from an increased risk of SCD other major findings in PCD include cardiomyopathy in especially children as well as hepatic failure and the most common symptom fatigue. Patients are treated with daily oral L-carnitine supplementation to prevent development of complications^4^.

Although PCD can cause lethal cardiac arrhythmia current treatment strategy does not include the use of an implantable cardioverter defibrillator (ICD) as primary prophylaxis. It is unknown if asymptomatic patients treated with L-Carnitine could still be susceptible to cardiac arrhythmia because of undiagnosed myocardial damage and fibrosis due to carnitine deficiency.

The aim of this study was to evaluate and compare cardiac function and myocardial scarring as late gadolinium enhancement with CMR in PCD patients with carriers and healthy subjects in order to assess if current prophylactic treatment strategy should be reevaluated.

## Methods

Sixty-eight subjects (18–75 years; 48 men) were included in the present study. Thirty-six patients with two different PCD genotypes were included: 17 patients homozygous for the c.95 A > G mutation and 17 patients compound heterozygous for c.95 A > G and a risk-haplotype (RH) in *SLC22A5*. Seventeen carriers of a PCD related mutation in *SLC22A5* and 17 healthy subjects were also included (Table 1).

**Table 1:** Baseline characteristics.

	c.95 A > G carriers n = 17	c.95 A > G/RH n = 17	c.95 A > G/c.95 A > G n = 17	p-value
Mean age (years)	35 (13)	38 (13)	32 (14)	0.44
Mean age at CTD diagnosis	29 (13)	33 (13)	26 (15)	0.20
Gender				
Men	12 (71%)	13 (77%)	12 (71%)	0.91
Women	5 (29%)	4 (24%)	5 (29%)	
BMI (kg m^−2^)	28 (5)	26 (5)	28 (5)	0.44
plasma L-carnitin, mmol/L	20.6 (7.8)	19.0 (8.2)	18.3 (9.6)	0.73

Data are presented as n (%) or mean ± standard deviation. c.95 A > G carriers, heterozygote; c.95 A > G/RH, compound heterozygotes for c.95 A > G and the risk haplotype; c.95 A > G/c.95 A > G, homozygote. P-values are from an ANOVA analyses or the χ² test as relevant. A p-value < 0.05 are considered significant.

The number of identified adult PCD patients homozygous for the c.95 A > G mutation alive in the Faroese population was 26 – of whom two had migrated, two had an implantable cardiac defibrillator (ICD) and therefore excluded, two patients declined to participate due to personal issues at the time and three patients suffered from claustrophobia.

All PCD patients received daily oral L-carnitine supplementation and were monitored in an outpatient setting with regular blood carnitine measurements. Carriers of a PCD related mutation are not treated with L-carnitine.

### Ethics, consent and permissions

All participants were informed orally and in writing and gave informed written consent before participation. The Faroese Ethics Committee and the Data Protection Agency approved the study and the study was carried out in accordance with the Helsinki Declaration.

### Consent to publish

Written consent to report individual patient data was obtained from participants if applicable.

### Mutations and genotypes

Patients were included from a nationwide screening program in the Faroe Islands. Individuals with blood levels of free carnitine below a lower cut-off level of 5 micromol/L were genetically tested for mutations related to PCD^3^. In a previous study, patients homozygous for the missense mutation c.95 A > G mutation in *SLC22A5* were shown to have only a mean residual OCTN2 transporter activity of 4% of normal, while patients compound heterozygous for the c.95 A > G mutation and the risk-haplotype (RH) have an 18% mean residual OCTN2 transporter activity^9^. The PCD carriers included in the study were carriers of the c.95 A > G mutation and were in the previous study shown to have a mean residual OCTN2 transporter activity of 46% compared to individuals without PCD^9^.

### CMR acquisition and analysis

**Figure 1:**
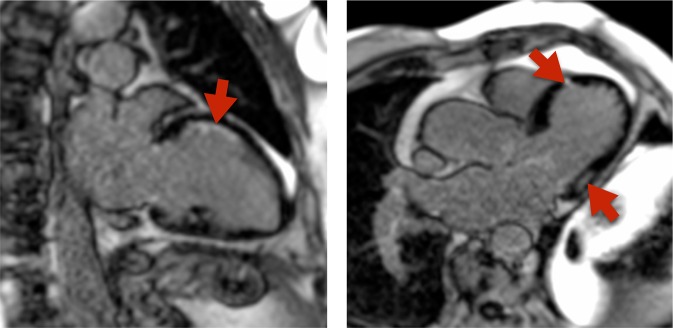
Cardiac magnetic resonance (CMR) examples of cardiac fibrosis involvement in primary carnitine deficiency (PCD). Arrows shows late gadelineum involvement representing cardiac fibrosis.

All patients underwent a CMR scan to evaluate left ventricular (LV) volumes, mass, systolic and diastolic function and scar tissue. CMR was performed on a 1.5 T scanner (Siemens, Aera, Erlangen, Germany) with a dedicated cardiac coil. LV volumes and mass were assessed using a steady-state free precession cine sequence (slice thickness 8 mm, no gap, echo time 1.5 ms, field of view 300–360 mm, phases 25). Multiple slices in both the short-axis and axial imaging planes were obtained covering the entire cardiac fossa. LV volumes were determined in the entire cardiac cycle (25 phases corresponding to a temporal resolution of 25–45 ms) using semi-automated endocardial contour detection with papillary muscles included in myocardial mass. Based on identification of LV myocardium, the part of the basal slice belonging to the LV was included, and the LV outflow tract was included based on reference to the 3-chamber image^10^. Stroke volume (SV) was end-diastolic volume (EDV) minus end-systolic volume (ESV). LV ejection fraction (LVEF) was SV divided by EDV (%). All volumes are presented as indexed to body surface area (BSA). Scar tissue was assessed using a delayed enhancement inversion-recovery sequence (slice thickness 8 mm, echo time 1.4 ms, field of view 300–360 mm, no slice gap; Fig. 1). Images were obtained 10 minutes after administration of diethylenetriamine pentaacetic acid (0.15 mL/kg; Gadovist, Bayer Schering, Berlin, Germany). Myocardial scarring was defined as enhanced myocardium in the LGE short-axis image sequences (>5 standard deviations above the intensity of normal myocardium; Fig. 1). LGE images was evaluated visually by two board certified CMR analyzers and disagreements were settled by consensus (PLM and NV). All other analyses were performed by one observer, blinded to all clinical data. All image processing was performed with dedicated software (CVI42 v. 4.0.1, Circle Cardiovascular Imaging, Calgary, Canada).

CMR with steady-state, free-precession sequences is considered the gold standard of cardiac chamber volumes (17). CMR is the method of choice for quantification of scar tissue compared with single-photon emission computed tomography owing to superior reproducibility and spatial resolution (18–20). Intra- and inter-observer variability of LVEDV, LVESV, left atrial maximum volume has been tested by our group in another study. Intra- and interobserver variability was lower than 2.86% for all measures^11^.

### Statistics

All continuous variables are reported as mean ± standard deviation (SD) and indexed to BSA (Mosteller´s formula) where appropriate. Binomial variables were expressed as numbers (%) and calculated differences between proportions with the χ² test. Normal distribution was tested visually on a histogram of the residuals and differences between group means or medians were assessed using ANOVA and post-hoc analyses using Tukey’s *post-hoc* test. Correlation was evaluated with Spearman’s correlation coefficient. All CMR values were compared to published published 95% confidence interval^12^. P value (double-sided) <0.05 was considered significant. All statistical analyses were performed using R (version 3.0.3; R Development Core Team 2014, http://www.R-project.org/) and GraphPad Prism 6.0c (GraphPad software inc., La Jolla, California, USA).

## Results

There were no differences between the groups of subjects with regards to age, sex, body mass index (BMI) and plasma L-carnitine (Table 1). Mean age at diagnosis of c.95 A > G homozygous PCD was 26 (15) years with no difference between the groups (p = 0.20). We included 71% (N = 17) of the identified adult c.95 A > G homozygous PCD patients in the Faroese population (N = 26).

Two c.95 A > G homozygous PCD patients had unexplained myocardial LGE (Fig. 1). One patient had LGE in the midmyocardium in the basal inferolateral segments, and the other patient had LGE in the midmyocardium in the lateral midventricular segment. A third elderly c.95 A > G homozygous patient had subendocardial LGE (50% transmurality) in the anterior segments, however the pattern was consistent with his history of previous myocardial infarction, and hence was not included in the analysis. There were no findings of myocardial LGE in any of the other patients, carriers nor healthy controls (p = 0.10).

**Figure 2:**
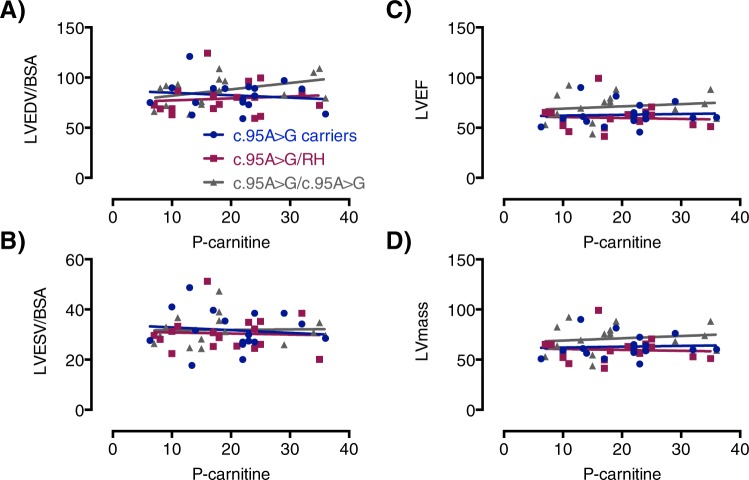
Plots showing the relation between (**A**) left ventricular end diastolic volume (LVEDV) and plasma carnitine levels (p-carnitine), (**B**) left ventricular end systolic volume (LVESV) and plasma carnitine levels (p-carnitine) and (**C**) left ventricular ejection fraction (LVEF) and plasma carnitine levels (p-carnitine). (**D**) left ventricular mass (LV mass) and plasma carnitine levels (p-carnitine). c.95 A > G carriers, heterozygote; c.95 A > G/RH, haplozygote; c.95 A > G/c.95 A > G, homozygote.

LVEDV, LVESV were not significantly different between groups, but LV myocardial mass (p = 0.037) was higher in c.95 A > G homozygous PCD patients (Table 2). Only one carrier subject and one c.95 A > G homozygous PCD patient had LV myocardial mass above the published 95% confidence interval (Table 3). All other parameters were comparable to published normal values for the left heart (Table 3). There were no significant correlations between P-carnitine and LVEDV/BSA, LVESV/BSA, LVEF or LV mass (Fig. 2).

**Table 2:** Data from Cardiovascular magnetic resonance imaging.

	c.95 A > G carriers n = 17	c.95 A > G/RH n = 17	c.95 A > G/c.95 A > G n = 17	p-value
Heart rate (min-1)	70 (12)	71 (15)	64 (11)	0.21
LVEDV (mL m^−2^)	82 (15)	79 (16)	87 (14)	0.30
LVESV (mL m^−2^)	32 (8)	30 (7)	32 (6)	0.82
LVSV (mL m^−2^)	51 (9)	49 (11)	55 (11)	0.16
LVCI (L min-1 m^−2^)	3.5 (0.5)	3.4 (0.9)	3.5 (0.7)	0.96
LVEF (%)	62 (5)	61 (5)	63 (5)	0.52
LV mass (g m^−2^)	63 (11)	60 (13)	71 (14)	0.037
LV Peak Ejection Rate (ml s^−1^):	567 (100)	529 (152)	581 (139)	0.50
LV Peak Filling Rate (ml s^−1^):	537 (108)	501 (150)	616 (146)	0.05
LV Average wall thickening (%)	71 (13)	77 (16)	76 (17)	0.47
LV Average wall motion (mm)	8 (1)	8 (1)	9 (2)	0.09
Presence of fibrosis, n	0 (0%)	0 (0%)	2 (12%)	0.12

Data are presented as n (%) or mean ± standard deviation. LV, left ventricle; LVEDV, left ventricular end-diastolic volume; LVESV, left ventricular end-systolic volume; LVSV, left ventricular stroke volume; LVEF, left ventricular ejection fraction; c.95 A > G carriers, heterozygote; c.95 A > G/RH, compound heterozygotes for c.95 A > G and the risk haplotype; c.95 A > G/c.95 A > G, homozygote. P-values are from an ANOVA analyses or the χ² test as relevant. A p-value < 0.05 are considered significant.

**Table 3:** Comparisons to normal values for left heart parameters^12,31^.

	c.95 A > G carriers n = 17	c.95 A > G/RH n = 17	c.95 A > G/c.95 A > G n = 17
LVEDV/[mean] n > 95% CI	1.05 (0.19) 2 (12%)	1.01 (0.21) 3 (18%)	1.12 (0.18) 5 (29%)
LVESV/[mean] n > 95% CI	1.22 (0.31) 5 (29%)	1.17 (0.28) 2 (12%)	1.22 (0.22) 3 (18%)
LVSV/[mean] n > 95% CI	0.97 (0.17) 1 (6%)	0.94 (0.21) 1 (6%)	1.06 (0.21) 3 (18%)
LVCI/[mean] n > 95% CI	1.06 (0.17) 2 (12%)	1.04 (0.26) 1 (6%)	1.05 (0.22) 4 (24%)
LVEF/[mean] n < 95% CI	0.92 (0.07) 4 (24%)	0.92 (0.08) 3 (18%)	0.94 (0.07) 3 (18%)
LV mass/[mean] n > 95% CI	0.91 (0.17) 1 (6%)	0.87 (0.19) 0	1.03 (0.20) 1 (6%)
LV Peak Filling Rate/[mean] n > 95% CI	1.07 (0.22) 0	1.00 (0.30) 0	1.23 (0.29) 0

Data are presented as n (%) or mean ± standard deviation. Comparisons include ratios to published means and 95% percentiles and numbers (%) above respective percentiles. CI, confidence intervals; [mean], published mean value; LV, left ventricle; LVEDV, left ventricular end-diastolic volume; LVESV, left ventricular end-systolic volume; LVSV, left ventricular stroke volume; LVEF, left ventricular ejection fraction; c.95 A > G carriers, heterozygote; c.95 A > G/RH, compound heterozygotes for c.95 A > G and the risk haplotype; c.95 A > G/c.95 A > G, homozygote. *P < 0.05. A p-value < 0.05 are considered significant.

## Discussion

Our main finding was that PCD patients homozygous for the severe c.95 A > G mutation had significantly higher LV mass and LV wall thickness compared to the other groups – with two of them exhibiting signs of unexplained myocardial scarring (12.5%) (Table 1). We found no difference in the overall cardiac function among the groups studied. To our knowledge, we are the first to systematically investigate a population of PCD patients for myocardial scarring and function using CMR.

The incidence of PCD in The Faroe Islands is the highest recorded in the world (1:297)^9^ and to our knowledge, we are the first to systematically investigate a population of PCD patients for myocardiac scarring and function using CMR. PCD patients can present with symptoms of severe hypoketotic hypoglycaemia, hepatic dysfunction, cardiac arrhythmias, cardiomyopathies or SCD^13–19^. However, a majority of the patients reach adulthood without any other symptoms than decreased stamina and fatigue^20,21^. The challenge is though that untreated – and perhaps treated - patients can be asymptomatic while still have an increased risk of SCD.

We have previously shown, that the hearts of newly diagnosed adult PCD patients were within normal echocardiographic ranges with no signs of structural abnormalities or cardiac hypertrophy – the results were though not stratified with regards to genotype^22^. CMR is superior to echocardiography to evaluate cardiac mass and dimensions, which could explain our novel finding of significantly higher mean LV mass in patients homozygous for the c.95 A > G mutation compared to the other groups. It also underpins our assumption that the mentioned genotype is associated with the most pronounced liability to develop symptoms in the Faroese PCD patient cohort.

We found unexplained myocardial scarring using LGE in two PCD patients homozygous for the c.95 A > G mutation. A previously published case report from the US also described areas of LGE in a patient with PCD who had been non-compliant with regards to L-carnitine supplementation^23^. To our knowledge, the incidence of areas of LGE in a hitherto healthy PCD population has not been investigated. Hence, we included a healthy control population in this study of whom none had any areas of LGE (n = 17). Neither had any of the carriers (n = 17) or patients suffering from the less severe genotype c.95 A > G/RH (n = 17). Our finding of two patients with unexpected myocardial scarring is noteworthy and gives rise to important questions.

The presence of LGE in patients with various cardiovascular diseases has been shown to predict adverse outcomes including ventricular tachycardia and ventricular fibrillation (VT/VF)^6,7^. A recent study in patients fulfilling the criteria for an implantable cardiac defibrillator (ICD) due to heart failure with reduced ejection fraction showed that patients with no areas of myocardial LGE on CMR were in a lower risk of VT/VF^24^. Myocardial scarring is thought to either induce or maintain ventricular arrhythmias in several ways: 1) scarring creates insulating barriers that form alterations in conduction in the fibrotic tissue, resulting in re-entrant wave fronts of excitation; 2) scarring can be considered as the origin or modulator of cardiac after-potentials that lead to triggered activity causing VT/VF; 3) oxidative and metabolic stress stimulate early after-depolarization; 4) in areas of myocardiac scarring the myocardium is less electrically connected and is hence more likely to initiate VT/VF (source-to-sink mismatches)^25–29^.

The relationship between myocardial scarring and VT/VF raises the question – are PCD patients with signs of myocardial scarring in an increased risk of SCD even when they adhere to their recommended L-carnitine supplementation? Should these patients receive a primary prophylactic ICD to prevent SCD? The questions are very relevant in this group of patients, because the primary prophylactic treatment strategy was e.g. used in the before mentioned case from the US, where the patient was fitted with an ICD. However, it needs to be stated that the US patient case was non-compliant to medical therapy. Three Faroese patients have also previously received ICDs.

Even though two of the 17 patients homozygous for the c.95 A > G mutation did have signs of myocardial scarring, our study indicates that it is not a very common finding, even in patients suffering from severe forms of PCD. Our experience gained during the last decade seems to indicate that the best way to prevent cardiac arrhythmia is treatment with L-carnitine supplementation as there have been no incidences of cardiac arrhythmia in Faroese PCD patients treated with L-carnitine. Adherence to the treatment has also proven to be very good in most Faroese patients, which has been verified by regular measurements of blood carnitine in all patients. PCD patients treated with L-carnitine in the Faroe Islands are thus not offered primary prevention with an ICD, instead they are, apart from being treated with L-carnitine, also closely monitored in an outpatient setting with regular blood tests and consultations with a clinical geneticist and cardiologist.

### Limitations

Although LGE is useful for reproducible assessment of myocardial scarring and replacement fibrosis, it is limited in its accuracy in the assessment of diffuse fibrosis. First of all, with conventional LGE imaging sequences, signal intensity is expressed on an arbitrary scale that is different in every image, and no direct comparisons can be made; second, the late enhancement is defined through contrast from a healthy area to an area with increased extracellular volume area and hence scarring or diffuse fibrosis cannot be assessed^30^. In a number of disease states, diffuse fibrosis can be assessed by quantitative CMR relaxometry, incorporating *T*_1_ mapping with high reproducibility, however it was not possible in the present study to include *T*_1_ mapping sequences.

## Conclusions

Some PCD patients have unexplained myocardial scarring shown by LGE. A relation between myocardial scarring, metabolic and oxidative stress and SCD has been shown in the literature. Hence, it can be speculated, that the myocardial scarring in some PCD patients combined with an oxidative or metabolic stress can be the substrate of the increased risk of SCD in untreated patients with PCD. L-carnitine supplementation is essential in order to prevent potentially lethal cardiac arrhythmia.

## Data Availability

The datasets used and/or analysed during the current study are available from the corresponding author on reasonable request.
